# Infection-induced increases to population size during cycles in a discrete-time epidemic model

**DOI:** 10.1007/s00285-024-02074-z

**Published:** 2024-04-10

**Authors:** Laura F. Strube, Shoshana Elgart, Lauren M. Childs

**Affiliations:** 1 Department of Mathematics, Virginia Tech, 225 Stanger St, Blacksburg, VA 24061 USA; 2 Department of Immunology, University of Pittsburgh Medical School, The Assembly, 5051 Centre Avenue, Pittsburgh, PA 15213 USA; 3 Department of Computational and Systems Biology, University of Pittsburgh Medical School, 800 Murdoch I building, 3420 Forbes Avenue, Pittsburgh, PA 15213 USA; 4 Laurel Springs School, 302 El Paseo Rd, Ojai, CA 93023 USA

**Keywords:** Discrete-time epidemic model, Bifurcation, Hydra effect, Overcompensatory growth, 92-10, 39-08

## Abstract

One-dimensional discrete-time population models, such as those that involve Logistic or Ricker growth, can exhibit periodic and chaotic dynamics. Expanding the system by one dimension to incorporate epidemiological interactions causes an interesting complexity of new behaviors. Here, we examine a discrete-time two-dimensional susceptible-infectious (SI) model with Ricker growth and show that the introduction of infection can not only produce a distinctly different bifurcation structure than that of the underlying disease-free system but also lead to counter-intuitive increases in population size. We use numerical bifurcation analysis to determine the influence of infection on the location and types of bifurcations. In addition, we examine the appearance and extent of a phenomenon known as the ‘hydra effect,’ i.e., increases in total population size when factors, such as mortality, that act negatively on a population, are increased. Previous work, primarily focused on dynamics at fixed points, showed that the introduction of infection that reduces fecundity to the SI model can lead to a so-called ‘infection-induced hydra effect.’ Our work shows that even in such a simple two-dimensional SI model, the introduction of infection that alters fecundity or mortality can produce dynamics can lead to the appearance of a hydra effect, particularly when the disease-free population is at a cycle.

## Introduction

A distinct feature of discrete-time models is their capacity to produce complicated dynamics in a single dimension (May 1974, 1987). The classic Ricker- (and logistic-) growth models, for example, are capable of producing a single stable fixed population size but also cyclic and chaotic behavior depending on the parameter values (May 1987; Anderson and May 1979). The capacity for such a range of behavior is the result of an overcompensatory growth term which exhibits peak per-capita reproduction at intermediate population sizes thereby allowing the system to repeatedly overshoot its equilibrium from above or below (Otto and Day 2007). In contrast, the saturating (compensatory) Beverton–Holt and Maynard–Smith–Slatkin growth forms produce a much simpler set of dynamics (Smith and Slatkin 1973; Bellows 1981; May et al. 1974; Hughes 2020). Discrete models with overcompensatory growth have a long history in the study of fisheries management as they capture features specific to the life history of fish (Ricker 1954; Beverton and Holt 1993). For example, many fish reproduce via seasonal spawning events in which reproductively mature individuals lay a large number of eggs which hatch weeks or months later and undergo a multistage process to maturity (Cushing 1969; Reid and Chaput 2012; US Fish and Wildlife Service 2023; State of Washington 2023; U.S. Fish and Wildlife Service 2023) and may be harvested seasonally (National Oceanic and Atmospheric Administration Fisheries 2023).

Historically a population is said to exhibit a ‘hydra effect’ if the population grows with increases in mortality (Abrams and Matsuda 2005; Sorenson and Cortez 2021; Abrams 2009). This paradoxical behavior was first identified by Ricker in 1954 when he used a discrete-time model to describe fishery populations (Ricker 1954; Abrams 2009). Since that time the hydra effect has been studied in a number of contexts using continuous-time ordinary differential equation (ODE) models, such as in (Abrams 2009; Abrams and Quince 2005; Abrams 2015; Abrams and Cortez 2015; Abrams and Matsuda 2005; Cortez and Yamamichi 2019; Cortez and Abrams 2016; Penczykowski et al. 2022; Adhikary et al. 2021; Sorenson and Cortez 2021). A few of these have gone beyond population models to focus on epidemiological dynamics (Penczykowski et al. 2022; Adhikary et al. 2021). Studies of discrete-time systems are more limited and most focus on population dynamics, many with harvesting (Cortez 2016; Cid et al. 2014; Franco and Peran 2013; Hilker and Liz 2013; Liz 2010, 2017; Liz and Ruiz-Herrera 2012; Jaramillo et al. 2022). A common feature of many of these models is overcompensatory growth, as the ‘hydra effect’ phenomenon is common when overcompensatory growth is coupled to a temporal separation of reproduction and mortality (Abrams 2009).

Expansion of one-dimensional population models through the introduction of additional processes, such as infection, further increases the complexity of system dynamics. Studies with two-compartment Susceptible-Infectious (SI) and Susceptible-Infectious-Susceptible (SIS) models have shown that disease can shift the bifurcation structure of a system and even introduce multi-stability (Castillo-Chavez and Yakubu 2001a, b; Castillo-Chavez and Yakabu 2002; Franke and Yakubu 2008; Xiang et al. 2021; Strube and Childs 2024). Increasing the dimension of a system also broadens possibilities for a hydra effect. For example in 2016, Cortez and Abrams in their study of discrete multispecies models (Cortez and Abrams 2016) generalized the definition of a hydra effect as a phenomenon in which population size increases due to the alteration of any parameter that directly reduces the fitness of a focal species. This was in contrast to the original definition that focused only on population increases due to changes in mortality (Abrams and Matsuda 2005). More recently, Abrams noted that indirect hydra effects are also possible through species interactions (Abrams 2019). That is, a mechanism which reduces the reproductive fitness of predators at an individual level might indirectly increase the size of the predatory population by causing an increase in a prey population.

Recently a second hydra-effect definition has been identified as a result of differing sub-populations in an infectious disease model. Namely, an infection which reduces reproductive fitness of infected individuals, either by increasing their mortality or reducing their fecundity, can increase the total population size (relative to the size of the corresponding disease-free system) (Jaramillo et al. 2022). Jaramillo et al. showed that disease-induced mortality is insufficient to produce such an infection-induced hydra effect from a stable fixed point of an SI system, but infection-induced reduction in per-capita reproduction is capable of producing this phenomenon. Jaramillo et al. (2022). Whether and how infection alters the total population size of an SI system when the disease-free system exhibits cyclic or aperiodic behavior remained an open question.

Here, we focus on a simple discrete-time model with Ricker growth, where the population is divided by disease status: susceptible and infectious. We consider scenarios in which disease may alter not only mortality but also reproductive capacity. Our system is identical to the form explored in Jaramillo et al. (2022) and is a simplification of a more complicated model incorporating virus and infectious host transmission, originally proposed to describe Salmon Anemia Virus (van den Driessche and Yakubu 2019; Yakubu 2020; Milliken and Pilyugin 2016). In these motivating models, disease transmission is assumed to be horizontal, which is in line with the current, albeit debated, consensus that vertical transmission of Salmon Anemia Virus is either non-existent or contributes minimally to the spread of disease (ISA 2007; Spickler 2011; Nylund et al. 2019; Christiansen et al. 2021). An argument for the reduction in reproductive output is based on the proposal that fish viruses which influence female health may alter fecundity through the production of lower quality of eggs and reduced hatchling survival (Kane-Sutton et al. 2010). Thus, in the SI model examined here, infection alters the population in two key ways: reduction in fecundity through reduced contribution in the Ricker function and decrease in the infectious class due to disease-induced mortality.

We find that the total population size exhibits counter-intuitive increases in response to factors, such as increase in mortality or reduction in fecundity, which reduce individual fitness. Furthermore, we find that the interplay between these two infection-induced effects and the already complicated dynamics produced by overcompensatory growth result in significant infection-induced changes in the location and types of bifurcations relative to the disease-free system. To examine these phenomena, we use numerical bifurcation analysis to show this system may exhibit two types of hydra effect: a “classical" hydra effect in which the total population size grows in response to an increase in a parameter which reduces individual fitness, and an "infection-induced" hydra effect in which the with-disease population is larger than the corresponding disease-free population for a specific set of model parameters. We analytically derive conditions sufficient to produce a hydra effect out of an infection-endemic equilibrium using each of these definitions. We show that the degree and category of hydra effect depends on initial conditions such than population increases can occur in two distinct ways: through continuous shifts in a bifurcation structure or through ‘jumps’ to a different stable structure in regions of parameter space which exhibit multi-stability.

## Model and methods

### Epidemiological models

**Fig. 1:**
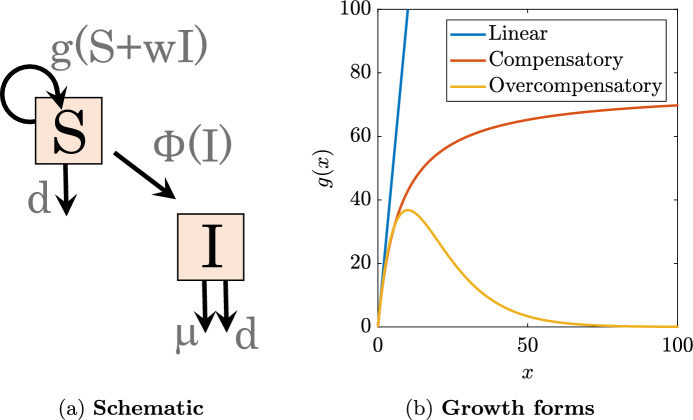
A discrete-time SI model with Ricker growth. **a** Schematic of the with-disease model. **b** Three examples of the growth function, *g*(*x*)

We consider two distinct discrete-time systems with the general forms,

$$\begin{aligned} {\bar{S}}_{t+1}&= g({\bar{S}}_t)+(1-d){\bar{S}}_t, \end{aligned}$$

and

$$\begin{aligned} S_{t+1}&= g(S_t+w I_t)+(1-d)S_t\phi (I_t), \\ I_{t+1}&=(1-d)S_t\left( 1-\phi (I_t)\right) + (1-d)(1-\mu )I_t, \end{aligned}$$

where *S* (or ${\bar{S}}$) refers to the susceptible population and *I* the infectious population. In these systems, *g*(*x*) is the reproduction term, *d* and $\mu$ are natural- and disease-induced probabilities of mortality in a given generation, respectively, and 1-$\phi (I)$ is the probability of infection in a given generation. The first system thus describes growth of a population, ${\bar{S}}$, as a function of natural reproduction and mortality in the absence of infection. The second system expands this model to include epidemiological dynamics, i.e., infection of susceptible individuals and disease-induced mortality of infected individuals (Fig. 1a).

We consider a scenario in which reproduction is of the Ricker form, $g(x)=rx\textrm{e}^{-bx}$ with growth parameter *r* and density-dependence *b*; infection occurs via a Poisson process, $\phi (I)=\textrm{e}^{-\beta I}$ at rate $\beta$ (such that $1-\phi (I)$ is the probability of the occurrence of at least one infection event); and disease influences the total population by disrupting reproduction, *w*, and increasing mortality, $\mu$. Thus the full form of the disease-free system is given by

1 $$\begin{aligned} {\bar{S}}_{t+1} = r{\bar{S}}_{t} \textrm{e}^{-b({\bar{S}}_{t})}+(1-d){\bar{S}}_{t}, \end{aligned}$$

the with-disease system by

2 $$\begin{aligned} S_{t+1}&= r(S_t+wI_t) \textrm{e}^{-b(S_t+wI_t)}+(1-d)S_t \textrm{e}^{-\beta I_t},\nonumber \\ I_{t+1}&=(1-d)S_t\left( 1-\textrm{e}^{-\beta I_t}\right) + (1-d)(1-\mu )I_t, \end{aligned}$$

and the total with-disease population by

3 $$\begin{aligned} T_{t+1}= r(S_t+wI_t) \textrm{e}^{-b(S_t+wI_t)}+(1-d)S_t + (1-d)(1-\mu )I_t. \end{aligned}$$

The Ricker-growth term is characterized as a type of overcompensatory growth that is linear for small populations, attains a maximum, and then decays exponentially as the population grows (Fig. 1b). This is in contrast to linear growth, which exhibits constant growth for all population sizes, and compensatory growth, which asymptotes to a maximum as population grows. Ricker growth can be tuned by two parameters: *r* and *b*. The reproduction capacity *r* describes the maximum per-capita growth and stretches the Ricker growth curve vertically. The factor *b* determines the extent of density-dependence and, thus, the scale on which growth occurs. The smaller the value of *b*, the larger the range of populations sizes with non-trivial reproduction, or put another way, decreasing *b* increases the population size at which the maximum per-capita reproduction occurs.

A key feature of discrete-time models is the implicit assumption that events occur in a particular order (Bodine et al. 2012). In our model, individuals reproduce with capacity $g(S+wI)$ before experiencing natural mortality, and the offspring they produce do not experience natural mortality until they are included in the population at the next time step. Infection occurs, with probability $1-\phi (I)$, to susceptible individuals that survive the interval (with probability $1-d$). Infected individuals, who survive natural mortality, can be lost due to disease-induced mortality, $\mu$. Individuals that become infected are not infectious and do not succumb to disease-induced mortality until the following generation. For clarity, we shift notation moving forward and discuss the effect of reducing fecundity, defined by ${\tilde{w}}=1-w$, on the behavior of the system. In this way, increases in either $\mu$ or ${\tilde{w}}$ reduce the individual fitness of infected individuals and may induce a hydra-effect.

We use ‘disease-free system’ to refer to Eq. (1) and ‘with-disease’ system to refer to System (2). It is important to note that these descriptors refer to the systems and not the state of the system at a given moment of time. That is, the with-disease system can experience infection extinction. Baseline parameters are described in Table 1 and were chosen for consistency with previous work (van den Driessche and Yakubu 2019; Jaramillo et al. 2022).

**Table 1:** Baseline model parameters

Parameter	Description	Baseline value
*r*	Maximum per-capita growth per generation	$\textrm{e}^4 \approx 54.6$
*b*	Scaling parameter for density-dependent growth	0.1
*d*	Probability of death due to natural causes	0.5
$\beta$	Poisson constant for infection transmission	0.056
*w*	Relative reproduction capacity of infected individuals	1
${\tilde{w}}$	Fecundity reduction factor (${\tilde{w}}=1-w$)	0
$\mu$	Probability of death due to infection	0

Values for *r*, *b*, *d*, $\beta$ from van den Driessche and Yakubu (2019)

### Simulation methods

Simulation and analysis was conducted in MATLAB 2021b and MATLAB 2023a. The disease-free system was simulated with the single Eq. (1) while the with-disease system was simulated with two-equation System (2). Details on construction of each figure is described in Appendix A. Codes are available at https://github.com/laurenchilds/SIdiscrete.

### Hydra effect definitions

In our analysis we consider two distinct hydra effect definitions. In the first definition, a population is said to exhibit a“classical” hydra-effect if the total population size increases as a parameter reducing individual fitness is increased. In the second, a population is said to exhibit an “infection-induced” hydra-effect if the total population size of the with-disease system is greater than that of the corresponding disease-free system for a fixed set of parameters.

#### Classical hydra effect for an SI system

The classical definition of a hydra effect was previously described by Cortez (2016) for a single compartment describing each species. To derive a definition for our two-compartment system, we rewrite the fixed point for the System (2) in the form

4 $$\begin{aligned} \begin{aligned} S^{*} = f_S(S^{*}(\alpha ), I^{*}(\alpha ), \alpha ) \\ I^{*} = f_I(S^{*}(\alpha ), I^{*}(\alpha ), \alpha ), \end{aligned} \end{aligned}$$

with emphasis on the dependence of the generic $\alpha$, related to fitness.

We say that a classical hydra effect exists at a fixed point if

5 $$\begin{aligned} \frac{\partial T^*}{\partial \alpha } = \frac{\partial S^*}{\partial \alpha } + \frac{\partial I^*}{\partial \alpha } > 0, \end{aligned}$$

i.e., if an increase in $\alpha$ (reduction in individual fitness) results in an increase in the total population size at a fixed point.

We say that a classical hydra-effect exists at a *k*-cycle if

6 $$\begin{aligned} \frac{1}{k}\sum _{i=1}^k\frac{\partial T_i^*}{\partial \alpha } = \frac{1}{k}\sum _{i=1}^k\left( \frac{\partial S_i^*}{\partial \alpha } + \frac{\partial I_i^*}{\partial \alpha }\right) > 0, \end{aligned}$$

where ($S^*_i$, $I^*_i$) is the *i*th fixed point in a *k*-order cycle of the with-disease system.

Note that for *k*-cycles the existence of a classical hydra effect is defined based on the average population size over time. Periodic or chaotic regimes may exhibit generation-to-generation declines across some time intervals but if on average the population size increases with respect to $\alpha$ the population is said to exhibit a classical hydra effect.

Here, we focus on $\alpha$ given by the fecundity reduction parameter (${\tilde{w}}$) or the disease-induced mortality parameter ($\mu$).

#### Infection-induced hydra effect for an SI system

The second formulation of a hydra effect was previously defined by Jaramillo et al. (2022). It is present if there exists a fixed point $(S^*, I^*)$ of System (2) such that

7 $$\begin{aligned} S^* + I^* > {\bar{S}}^*, \end{aligned}$$

where

$$\begin{aligned} {\bar{S}}^* = \frac{1}{b} \ln \left( \frac{r}{d} \right) \end{aligned}$$

is a fixed point of System (1).

For higher order behavior, an infection-induced hydra effect occurs if

8 $$\begin{aligned} \frac{1}{k}\sum _{i=1}^{k} \left( S_i^* + I_i^*\right) > \frac{1}{{\bar{k}}}\sum _{i=1}^{{\bar{k}}}{\bar{S}}_i^*, \end{aligned}$$

where ($S_i^*,I_i^*$) is the *i*th fixed point in a *k*-order cycle of the with-disease system and $\bar{S_i}^*$ is the *i*th fixed point in a ${\bar{k}}$-order cycle of the disease-free system.

Note we calculate an average population size of the with-disease and disease-free system independently. That is *k* and ${\bar{k}}$ need not be identical.

## Analytical results

In this section we derive sufficient conditions for the existence of a classical hydra effect and for an infection-induced hydra effect at a fixed point of System (2). All proofs to Theorems and Lemmas are found in the Appendix B.

### Classical hydra effect at a fixed point of an SI system

From the fixed point ($S^*,I^*$) there is a hydra effect with increases in $\alpha$ if

$$\begin{aligned} \frac{\partial T^*}{\partial \alpha } = \frac{-\left( \left( \frac{\partial f_I}{\partial I}-1\right) \frac{\partial f_S}{\partial \alpha } -\frac{\partial f_S}{\partial I}\frac{\partial f_I}{\partial \alpha } -\frac{\partial f_I}{\partial S}\frac{\partial f_S}{\partial \alpha } + \left( \frac{\partial f_S}{\partial S} -1\right) \frac{\partial f_I}{\partial \alpha }\right) }{|{\textbf{J}}-\mathbf {I_2}|} \Bigg |_{(S^*,I^*)} >0. \end{aligned}$$

where ${\textbf{J}}$ is the Jacobian of System (4). A derivation of this definition can be found in Appendix B.6.

### Sufficient conditions for the classical hydra effect with changes in mortality

#### Theorem 1

Given System (2) with fixed point $T^*$, if $\ln \left( \frac{r}{d}\right) <\frac{wbd}{\beta (1-d)}$ and there is a hydra effect when $\mu$ is the hydra effect parameter, i.e.,

$$\begin{aligned} \frac{\partial T^*}{\partial \mu }>0, \end{aligned}$$

then there is also a hydra effect when ${\tilde{w}}$ is the hydra effect parameter, i.e.

$$\begin{aligned} \frac{\partial T^*}{\partial {\tilde{w}}}>0. \end{aligned}$$

The proof of Theorem 1 is found in Appendix B. To prove this, we use the existence and uniqueness of the endemic equilibrium (Lemma 4, stated in Sect. 3.3 and proved in Appendix B.5) as well as the following lemma.

#### Lemma 2

Considering System (2), the unique endemic equilibrium $(S^*, I^*)$ satisfies

$$\begin{aligned} S^* + w I^* < {\bar{S}}^*. \end{aligned}$$

The proof of Lemma 2 is found in Appendix B.3.

### Sufficient conditions for infection-induced hydra effect

A sufficient set of conditions for the increase in total population size originating from a fixed point in the disease-free system is found in Theorem 5. In order to derive these conditions, we reformulate the fixed point of System (2) into a one-dimensional system in Lemma 3.

#### Lemma 3

(One-dimensional equivalent system) A fixed point of the System (2) is equivalent to a fixed point of the equation

9 $$\begin{aligned} \ln \left( 1-Cz\right) = z\big (\ln \left( A+Bz\right) - \ln (1+Dz)-D\ln (1-Cz)\big ), \end{aligned}$$

where

$$\begin{aligned} \begin{aligned} z&:= \frac{\beta I}{b S}, \quad A:= \frac{d}{r},\qquad B:= \frac{b(1-\nu )}{\beta r},\quad C:= \frac{b(1-\nu )}{\beta (1-d)}, \quad D:=\frac{bw}{\beta },\\&\textrm{and}\quad \nu :=(1-d)(1-\mu ). \end{aligned} \end{aligned}$$

The proof of Lemma 3 is found in Appendix B.4.

#### Lemma 4

The endemic equilibrium (satisfying $I^* > 0$) exists and is unique.

**Fig. 2:**
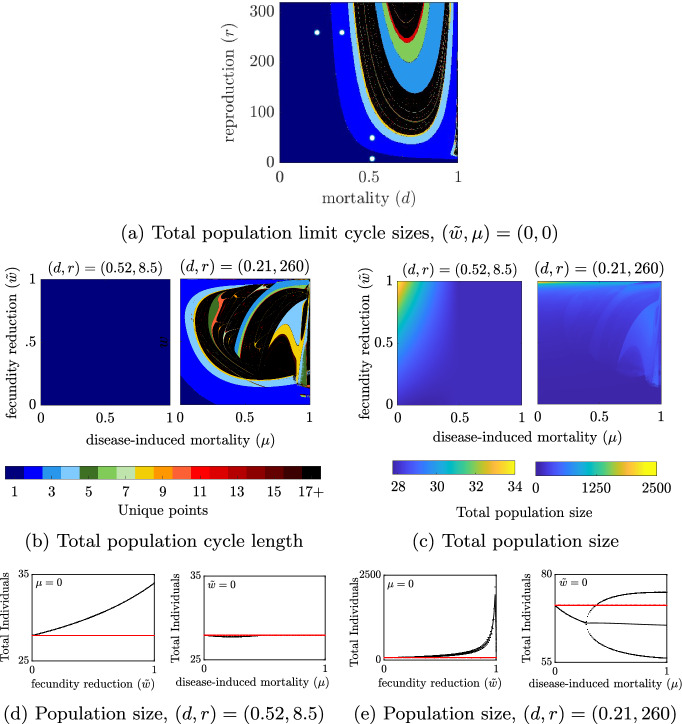
The influence of ${\mu }$ and ${\tilde{\textbf{w}}}$ on the bifurcation structure and total population size for $\mathbf {(d,r)}$ pairs selected from the fixed-point regime of the disease-free system. All parameters at baseline values except: *d*, *r* as indicated. For **a**–**c**, $\mu \in [0:0.001:1]$, ${\tilde{w}}\in [0:0.001:1]$. Simulations were completed with standard conditions except as follows: simulations with $(d,r)=(0.52,8.5)$ were completed with initial conditions $(S_0,I_0)=(150,20)$ and 50,000 transient generations, simulations with $(d,r)=(0.21,260)$ were completed with initial conditions $(S_0,I_0)=(60,20)$ and 10,000 transient generations. Points in **a**: $(d,r) = (0.52,8.5), (0.52, 50), (0.21,260), (0.35,260)$

The proof of Lemma 4 is found in Appendix B.5.

#### Theorem 5

(Hydra effect sufficient conditions) Given a fixed point $(S^*,I^*)$ for System (2), which is equivalent to a fixed point $z^*$ for the Eq. (B1), sufficient conditions to guarantee an increase in population size when infection is introduced (infection-induced hydra effect) are

$$\begin{aligned} w < 1 - \frac{\beta }{b} \quad \textrm{and} \quad d\ge \frac{1}{2}. \end{aligned}$$

We note that the existence of the hydra effect is independent of $\mu$.

The proof of Theorem 5 is found in Appendix B.6.

## Simulation results

In this system, disease acts on the population through two mechanisms which each reduce individual fitness: it reduces the fecundity of the infected population by *w* and increases the mortality of the infected population by $\mu$. For clarity, we report the influence of infection on fecundity using the *fecundity reduction factor*, ${\tilde{w}}=1-w$. This means that if ${\tilde{w}}=0$ $(w=1)$, infected individuals exhibit full reproductive capacity, and if ${\tilde{w}}=1$ $(w=0)$, they do not reproduce. The parameter $\mu$ is the probability of death due to disease. When $\mu =0$, the disease is never fatal; when $\mu =1$, there is a 100% chance of disease-induced mortality in a given generation.

When $(\mu ,{\tilde{w}})=(0,0)$ the bifurcation structure (Fig. 2a) is identical to that of the disease-free system. In this scenario, infection moves individuals into an infected class which exhibits reproduction and mortality identical to that of the susceptible class. Under this condition, the system exhibits a fixed-point for all mortality probabilities (*d*) when the reproduction (*r*) is low and for all per-capita reproduction (*r*) when the probability of mortality (*d*) is low. If *r* is increased above a threshold at $r\approx 7.0$, sufficiently large *d* can induce the presence of cycles, and if *d* is increased above a threshold at $d\approx 0.285$, sufficiently large *r* can induce the presence of cycles. Additionally, we observe what appears to be chaotic behavior for some regions of *d*, *r* parameter space, but do not distinguish chaotic behavior and $>16$-cycles in our analyses.

### Capacity of infection to alter the total population depends on the level of natural mortality and reproduction

We examine the long-time behaviors of a fixed-point in the total population dynamics for a broad range of mortality (*d*) - reproduction (*r*) parameter space when $(\mu ,{\tilde{w}})=(0,0)$ (Fig. 2a). In particular, the system exhibits fixed-point behavior at both $(d,r)=(0.52,8.5)$ and at $(d,r)=(0.21,260)$ (Fig. 2a, bottom and far-left white dots). However, the capacity of infection to alter the bifurcation structure of the system differs in these two cases. With intermediate mortality but low reproductive capacity, $(d,r)=(0.52,8.5)$, the system does not exhibit any bifurcations in response to changes in $\mu$ or ${\tilde{w}}$ (Fig. 2b). However, with low mortality and high reproductive capacity, $(d,r)=(0.21,260)$, the system shows that sufficient increase of either the fecundity reduction factor ${\tilde{w}}$ or the disease-induced mortality probability $\mu$ produces at least one period-doubling bifurcation point and entry into a cyclic regime and likely chaos.

While infection does not alter the bifurcation structure of the system at intermediate mortality and low reproductive capacity, $(d,r)=(0.52,8.5)$, examination of the total population size across $(\mu ,{\tilde{w}}) \in [0,1]\times [0,1]$ shows that it does alter the equilibrium total population size (Fig. 2c). In particular, increasing the disease-induced mortality, $\mu$, predictably reduces the total population size while reducing the fecundity of the infected population actually increases the total population (Fig. 2d). This is because the increased per-capita reproduction that results from a reduction in the reproducing population is more than sufficient to compensate for the loss due to disease-induced mortality. The reduction in population size with increases in disease-induced mortality at a fixed point is proven in Jaramillo et al. (2022).

Similarly, when $(d,r) = (0.21,260)$, a reduction in fecundity produces clear increases in the total population size in the absence of disease induced mortality $\mu =0$ (Fig. 2e). An increase in mortality decreases the total population size on average in the absence of effects on reproduction (${\tilde{w}})$. These qualitative behaviors are consistent with the $(d,r)=(0.52,8.5)$ case despite the fact that each parameter, $\mu$ and ${\tilde{w}}$, induce a period doubling bifurcation in the with-disease system.

### Infection-induced reduction in fecundity can induce a hydra effect

**Fig. 3:**
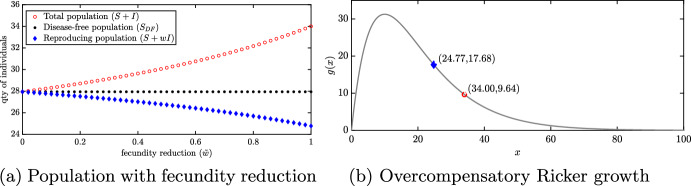
Influence of infection on reproduction via the fecundity reduction factor ${\tilde{w}}$. **a** Long-time total population counts for $(d,r)=(0.52,8.5)$ as ${\tilde{w}}$ varies with no disease-induced mortality, $\mu$=0. All parameters are at baseline values except: $(d,r)=(0.52,8.5)$ and ${\tilde{w}} \in [0:0.02:1]$. Standard simulation conditions with initial conditions $(S_0,I_0) = (150,20)$. **b** Ricker growth function, $g(x)=rx\textrm{e}^{-bx}$ with $r=8.5$ and $b=0.1$, as population size, *x*, changes. The blue triangle indicates the reproducing population size ($S+wI\approx 24.77$) and the red circle the total population ($S+I\approx 34.00$) in the with-disease system when ${\tilde{w}}=1$

With the introduction of infection, we find that the total population of the with-disease system often exceeds the total population of the disease-free system. Consider the total population with $(d,r)=(0.52,8.5)$. Increasing the fecundity reduction factor ${\tilde{w}}$ in the absence of disease-induced mortality, i.e. $\mu =0$, causes the total population size to increase (Fig. 3a). In particular, while the disease-free system exhibits a constant ${\bar{S}}\approx 27.94$ individuals independent of ${\tilde{w}}$, which has no impact on the disease-free population, the total population of the with-disease system grows with increases in ${\tilde{w}}$ even as its reproducing population declines (Fig. 3a). This is due to the overcompensatory nature of the Ricker growth function (Fig. 3b). When ${\tilde{w}}=1$, the total population in the with-disease system is $S+I\approx 34.00$ while the total reproducing population in the with-disease system is $S+wI\approx 24.77$. By reducing the size of the reproducing population, the disease actually increases the per-capita reproduction by a factor of $\sim 2$. This is consistent with analysis in Jaramillo et al. (2022), which showed that there is no possibility of infection-induced hydra effect from a endemic equilibrium without reductions to fecundity.

### Appearance of infection-induced hydra effect depends on the natural parameters for mortality and reproduction

**Fig. 4:**
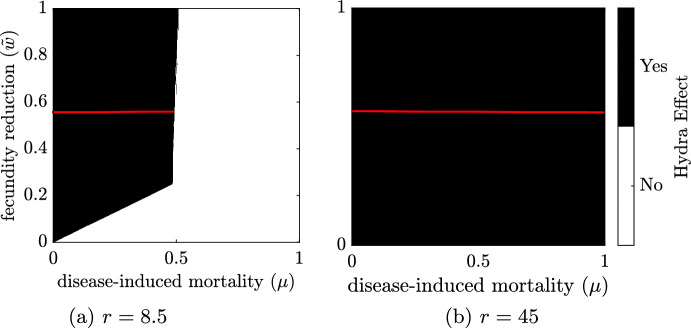
Regions of $({\mu },{\tilde{\textbf{w}}})$-parameter space exhibiting any degree of hydra effect. Parameters are at baseline values except $d=0.52$, *r* as indicated, $\mu \in [0:0.001:1]$ and $w \in [0:0.001:1]$. Standard simulation conditions combining ten sampled initial conditions: $(S_0,I_0)=$ (365, 726), (485, 61), (241, 432), (674, 530), (596, 956), (725, 298), (876, 167), (956, 658), (131, 873), and (64, 380). The area above red line indicates the sufficient condition for the hydra effect in Theorem 5. Here, the condition falls at ${\tilde{w}}=\frac{\beta }{b}=0.56$, given the existence of an endemic equilibrium

The capacity of infection to induce a hydra effect depends on the value of the natural parameters for mortality (*d*) and reproduction (*r*). For moderate mortality but low reproduction, $(d,r)=(0.52,8.5)$, a hydra effect is observed for all values of ${\tilde{w}}$ when $\mu =0$. For $\mu \in [0,0.5]$, a hydra effect is possible provided ${\tilde{w}}$ is above a threshold that increases with increasing $\mu$ (Fig. 4a). No hydra effect occurs for any value of ${\tilde{w}}$ if $\mu$ is above a threshold of $\mu \approx 0.5$, because the infection dies out and thus infection-induced hydra effect is not possible. In contrast, when reproduction is higher, $(d,r)=(0.52,45)$, a hydra effect is observed for all ${\tilde{w}}$ and $\mu$ (Fig. 4b). Our sufficient analytical conditions, found in Theorem 5, correspond to numerical simulations (Fig. 4, area above the red line).

### Sensitivity to initial conditions in the chaotic regions causes propagation of error

To quantify the hydra effect we rely on the numerically determined average population size. In regions with complicated dynamics this can be difficult due to numerical error. For example, within the chaotic regions, we observe divergence between what should be equivalent trajectories in the disease-free and with-disease systems. Mathematically, when there is no fecundity reduction ($${\tilde{w}}=0$$) and no disease-induced mortality ($$\mu =0$$) the equation for the total with-disease population is equivalent to that of the disease-free system, since $$ T_{t}= S_t+I_t$$. That is, Eq. (3) reduces to

$$\begin{aligned} T_{t+1}= rT_t \textrm{e}^{-bT_t}+(1-d)(T_t). \end{aligned}$$

**Fig. 5:**
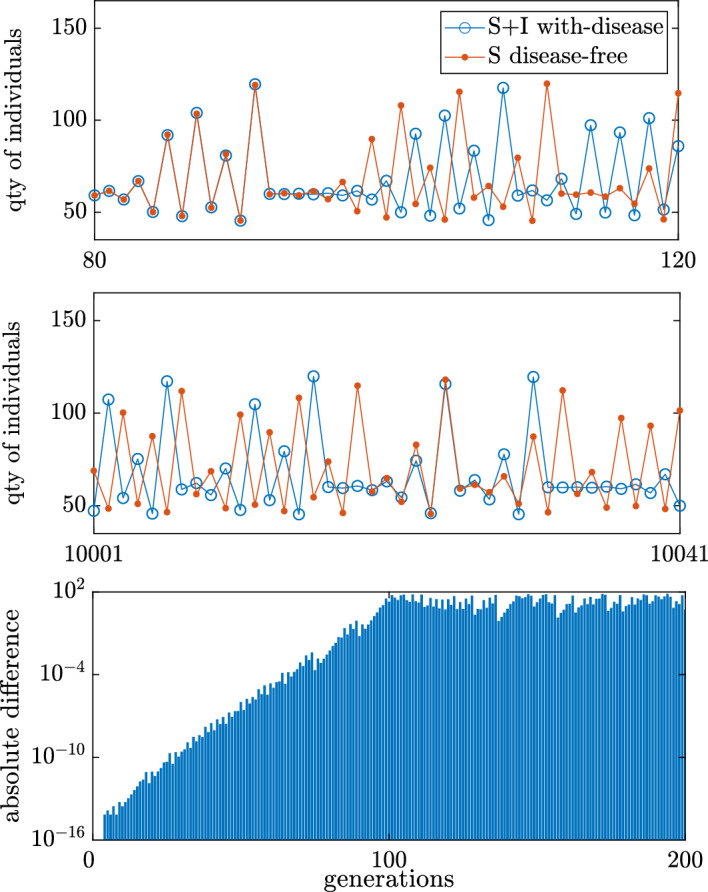
Disease-free and with-disease system trajectories originating in the chaotic regime. *Top: * behavior early in the simulation from generations 80–120. *Middle:* long-time behavior from generations 10,001–10,041. *Bottom:* log-linear plot of absolute difference between the disease-free and with-disease system trajectories. Parameters are at baseline values except: $r=200$ and $(\mu ,{\tilde{w}})=(0,0)$. Standard simulation conditions with initial conditions $(S_0, I_0) = (150, 20)$ for the with-disease system and ${\bar{S}}_0=170$ for the disease-free system

However, due to sensitivity to initial conditions in the apparent chaotic regions, it is possible for individual simulations to diverge over time due to the accumulation of numerical error. For example, when $(d,r)=(0.5,200)$ and $(\mu ,{\tilde{w}})=(0,0)$, the total population dynamics of the with-disease system visibly diverges from that of the disease-free system within 120 generations (Fig. 5, top). However, because the total population in the system is bounded, the difference between the measured total population sizes is also bounded. Visually this is apparent within 200 generations as the absolute difference rapidly rises from numerical precision over the first generations, but never exceeds 100 (Fig. 5, bottom). The average of the total population size of the disease-free system and the with-disease system will be numerically very similar if enough generations are averaged together. In practice, for $(\mu ,{\tilde{w}})=(0,0)$, which we know should have identical disease-free and with-disease total population size, we find that averaging 2000 generations is sufficient to get averages within reasonable agreement (See Appendix C).

### Initial conditions affect the total population bifurcation structure and hydra effect

**Fig. 6:**
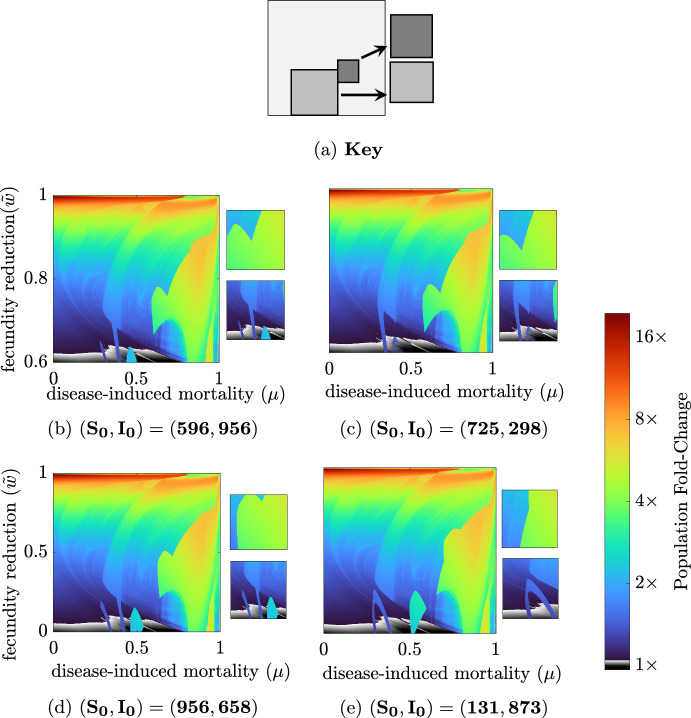
Infection-induced fold-change in total population with respect to $\mu$ and ${\tilde{w}}$ for four distinct pairs of initial conditions. (a) Key for each panel: Large diagram, $(\mu ,{\tilde{w}}) \in [0:0.001:1]\times [0:0.001:1]$; top small diagram $\mu \in [0.6:0.001:0.8]$ and ${\tilde{w}}\in [0.3:0.001:0.5]$; bottom small diagram $\mu \in [0.2:0.001:0.6]$ and ${\tilde{w}}\in [0:0.001:0.4]$. All parameters are at baseline values except $r=260$, $d=0.35$ and $(\mu ,{\tilde{w}})$ as stated above. Standard simulation conditions are used but initial conditions are varied as listed

Up to this point, results focused on a particular set of initial conditions: $(S_0,I_0)=(150,20)$. However, the long-time behavior is, at times, sensitive to the choice of initial conditions. To assess the potential variation based on initial conditions, we used Latin hypercube sampling of initial conditions across ten uniform intervals for $S_0 \in [0,1000]$ and $I_0 \in [0,1000]$. While simulations indicate a general consistency in the presence of hydra effect trends independent of initial conditions across wide regions of parameter space, there are parameter ranges in which initial conditions have significant influence (Fig. 6). The importance of initial conditions is most pronounced for small ${\tilde{w}}$, where the system exhibits mild hydra effect (less than 5% increase in population size) for most levels of disease-induced mortality, $\mu$ (Fig. 6, gray-scale). However these regions where there is no hydra effect or only small increases in population size are interrupted by regions of 2- and 4-fold increases in population size. The precise size and location of these large increases are determined by the initial conditions (Fig. 6).

**Fig. 7:**
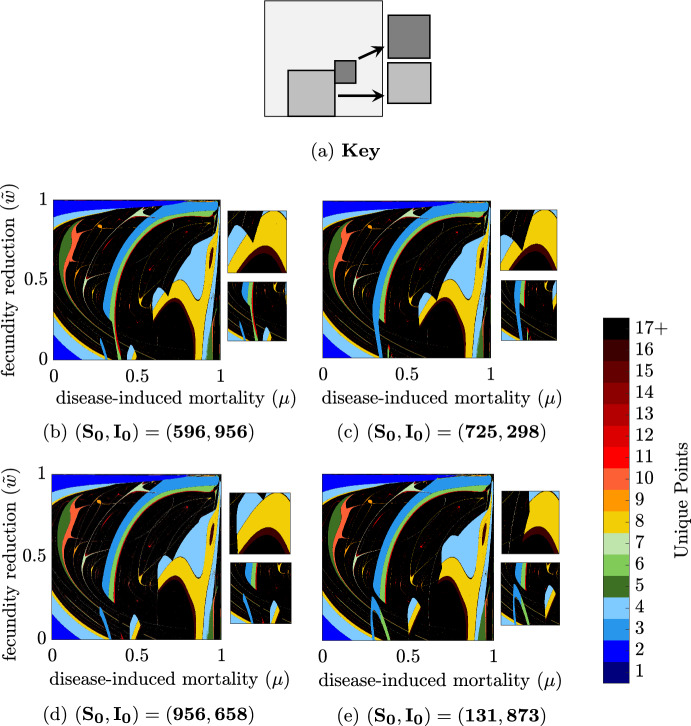
Infection-induced total population cycles for the with-disease system with respect to $\mu$ and ${\tilde{w}}$ for four distinct pairs of initial conditions. **a** Key for each panel: Large diagram, $(\mu ,{\tilde{w}}) \in [0:0.001:1] \times [0:0.001:1]$; top small diagram, $\mu \in [0.6:0.001:0.8]$ and ${\tilde{w}}\in [0.3:0.001:0.5]$; bottom small diagram, $\mu \in [0.2:0.001:0.6]$ and ${\tilde{w}}\in [0:0.001:0.4]$. Colorbar: black indicates a reduction in population; gray-scale indicates 0–5% increase in population over the disease-free system; color-scale indicates $>5$% increase in population, represented by fold-change. All parameters are at baseline values except $r=260$, $d=0.35$ and $(\mu ,{\tilde{w}})$ as stated above. Standard simulation conditions are used but initial conditions are varied as listed

The observed jumps in total population size, despite only small shifts in parameters, typically occur at that same point as sharp changes in the long-time behavior (Fig. 7), i.e. changes in the bifurcation structure. Furthermore, these changes in bifurcation structure show similar patterns when initial conditions are changed. As shown in Strube and Childs (2024), the system exhibits multiple potential long-time behaviors (bi- or multi-stability) for regions of parameter space. Which of these stable long-time behaviors is realized depends on the initial conditions used for a simulation. As the parameter space is large, we leave a full examination of initial conditions and multi-stability to future work.

### The introduction of infection allows for hydra effects through a continuous shift in bifurcation structure or via a jump between distinct structures

**Fig. 8:**
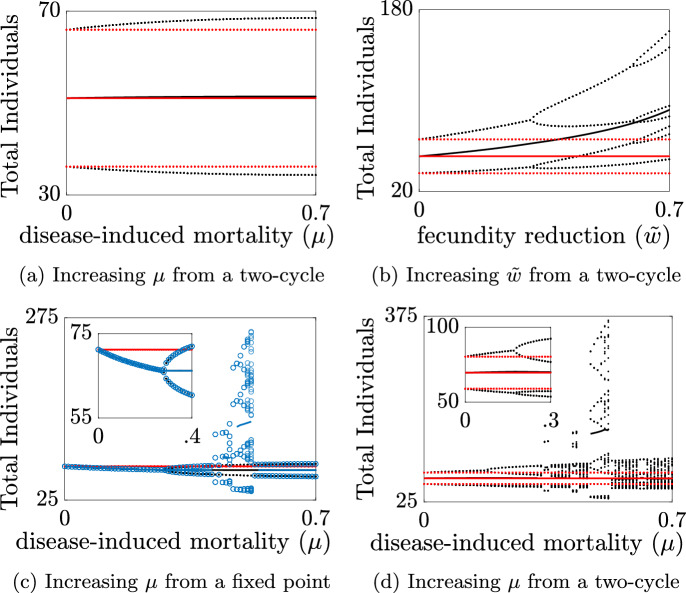
Examples of classical and infection-induced hydra effect. Classical and infection-induced hydra effect from **a** a disease-free two-cycle as disease-induced mortality increases, **b** a disease-free two-cycle as reduction in fecundity increases, **c** from a fixed point as disease-induced mortality increases, but only via jumping between structures, and **d** a disease-free two-cycle as disease-induced mortality increases. Black dots are long-time behavior of the disease-free system with the average population in a solid black line. Colored red dots in **a**–**d** and colored circles in **c** are long-time behavior of the with-disease system with the average population in a solid colored line. Standard simulation conditions with all parameters and initial conditions at baseline except: **a** $r=50$, $d=0.52$, ${\tilde{w}}=0$, $(S_0,I_0) = (150,20)$; **b** $r=50$, $d=0.52$, $\mu =0$, $(S_0,I_0) = (150,20)$; **c** $r=260$, $d=0.21$, ${\tilde{w}}=0$, $(S_0,I_0) = (60,20)$ in black, $(S_0,I_0)= (180,20)$ in blue; **d** $r=260$, $d=0.35$, ${\tilde{w}}=0$, $(S_0,I_0) = (956,658)$. **a**, **c**, **d** with $\mu \in [0,0.7]$, and **b** with ${\tilde{w}}\in [0,0.7]$

A close examination of our system shows both types of hydra effect (classical and infection-induced) with the introduction of infection (Fig. 8). The appearance of these increases in population size occur when the disease-free and with-disease systems both show identical types of long term behavior and when they show distinct types of long-time behavior. The latter may involve change along a single bifurcation structure or jumps between structures when multi-stability is present. To illustrate, we fix natural birth and mortality and vary a single disease parameter ($\mu$ or ${\tilde{w}}$) between 0 and 0.7. We focus on parameter sets in which the disease-free system exhibits a two-cycle, but also consider one example when the with-disease system bifurcates from a fixed point to a two-cycle before exhibiting a hydra effect (parameter sets shown in Fig. 2a, three upper white dots).

For $(d,r) = (0.52,50)$, and no reduction of fecundity with infection (${\tilde{w}}=0$), the with-disease system shows simple forms of classical and infection-induced hydra effects (Fig. 8a). The classical hydra effect is evident by the slight increase in the total population average as $\mu$ increases as a result of a infection-induced increase in the amplitude of the two-cycle that favors the upper branch. The infection-induced hydra effect is seen by the larger with-disease population size relative to the disease-free population at a given level of disease-induced mortality ($\mu >0$).

At these same *d* and *r* values, when infection reduces fecundity, but does not increase mortality ($\mu =0$), both types of hydra effect are more obvious (Fig. 8b). Here, infection shifts the cyclic behavior toward larger population sizes as ${\tilde{w}}$ is increased. The classical hydra effect is seen by increases in population size, left-to-right and the infection-induced hydra effect is seen by population increases bottom-to-top. Note that in this scenario the with-disease system bifurcates such that the cycle-size diverges from that of the disease-free system, such that $k\ne {\bar{k}}$ for the hydra effect definition in Sect. 2.3.2).

We showed analytically that without reductions in fecundity, and starting from a population endemic equilibrium, changes in $\mu$ are unable to produce a hydra effect (Theorem 1) in our discrete SI system. However, if other stable population structures exist, the introduction of infection can knock the population into the basin of attraction of another stable structure, which has a larger average population size. We refer to this shift between distinct stable long-time behaviors as “structure-jumping,” and can be seen in Fig. 8c for $(d,r) = (0.21,260)$ near $\mu =0.5$.

This structure-jumping is even more apparent for large $\mu$ values when there is no effect on fecundity, ${\tilde{w}}=0$ (Fig. 8d). In the absence of disease-induced mortality, $\mu =0$, or reductions in fecundity, we know the disease-free and with-disease systems have identical population size, and thus, no hydra effect is possible. When $(d,r)=(0.35,260)$ and disease-induced mortality increases from zero, the system first undergoes a period doubling bifurcation near $\mu \approx 0.179$ from a two-cycle to a four-cycle and again at $\mu \approx 0.315$ from a four-cycle to an eight-cycle. These bifurcations have minimal influence on the average population size. However, when $\mu$ reaches a threshold near $\mu \approx 0.337$ the long-time behavior jumps to a three -cycle before returning to what appears to be a continuation of the previous eight-cycle near $\mu = 0.352$. This structure undergoes additional period doubling en route to chaos as $\mu$ increases. During this progression the system exhibits additional jumps between structures, for example for $\mu$ approximately between 0.411 and 0.430 and again for $\mu$ approximately between 0.461 and 0.528. When these shifts in structure occur, there are clear differences in the average total population size of the disease-free and with-disease systems and the system clearly exhibits an infection-induced hydra effect.

## Discussion

Classically, a biological process which decreases the fitness of individuals but paradoxically increases the total population size is said to induce a hydra effect (Abrams 2009; Abrams and Matsuda 2005) and is seen in a variety of systems, e.g., (Sorenson and Cortez 2021; Cortez and Abrams 2016; Abrams 2009; Abrams and Cortez 2015; Abrams 2002; Abrams et al. 2003). Jaramillo et al. (2022) introduced a new hydra-effect definition, which they termed an infection-induced hydra effect, to describe scenarios in which the introduction of infection is capable of increasing total population size relative to the corresponding disease-free system, but without changes to any specific parameters. They found that such a population size increase does not always require increased mortality, but may also be caused by a reduction in the fecundity of infected individuals. In addition, multiple studies have shown that the addition of infection to a discrete-time model with Ricker growth can introduce multi-stability in the system (Castillo-Chavez and Yakubu 2001a, b; Castillo-Chavez and Yakabu 2002; Franke and Yakubu 2008; Xiang et al. 2021; Strube and Childs 2024).

In this work, we extend examination of the two-dimensional SI model with Ricker-growth by performing numerical bifurcation analysis and assessing the capacity of infection to increase total population size. In particular, we focus on parameter regions where the disease-free system exhibits complicated behavior such as cycles. We show that infection can increase total population size both in regions where the same type of long-time dynamics are seen for the disease-free and with-disease systems and when different long-time dynamics occur. We provide a set of sufficient analytical conditions for which the hydra effect is observed when the disease-free system is in an endemic equilibrium, which is consistent with our numerical simulations. We show that increases in disease-induced mortality or infection-induced reductions in fecundity are capable of altering the bifurcation structure with long-time persistence of infection.

Overall, we observe two distinct types of hydra effect in our SI system: ‘classical’ and ‘infection-induced’ (Fig. 8). In the classical case, we see increases in total population size with smooth changes to a parameter that tunes individual fitness. In the infection-induced case, we see population increases relative to the disease-free system without changes to any parameters.

For each of these two types of hydra effect, population increases can arise in two settings. Population increases can occur when the disease-free and with-disease systems have qualitatively similar long-time behaviors (i.e., both are fixed points, both are cyclic with the same cycle length, or both are chaotic) such that infection induces an overall shift in the total population and results in an average with-disease population size greater than the average disease-free population size. Population increases can also occur when the disease-free system and with-disease system exhibit different long-time behaviors. These behaviors may be part of the same bifurcation structure or from two distinct bifurcation structures, where we observe a ‘jump’ into a fundamentally distinct long-time behavior regime such that the average total population increases. This requires the presence of bi- or multi-stability in the system, such that multiple long-time behaviors are possible. When a qualitatively different behavior occurs, e.g., low-dimensional cycle vs chaotic behavior, the total population size can be drastically different.

When the disease-free system is in a stable fixed point, our novel analytic calculations, consistent with those of Jaramillo et al., show that infection-induced increases in population are not possible with increases in mortality unless there are also decreases to the fecundity of infected individuals. However, in contrast to Jaramillo et al., we show that a hydra effect can occur from a fixed point in the disease-free system if increases in a fitness parameter causes a jump between bifurcation structures (Fig. 8c). We also show that changes to disease-induced mortality are sufficient to produce hydra effects from regions of parameter space which produce cyclic or chaotic behavior in the disease-free system. The key feature here is the bifurcation to a cycle of order two or higher, such that enhanced mortality of the infected class can alter the fecundity across the full length of the cycle. For example, disease-induced mortality can increase population size from a two-cycle because it lowers the population sufficiently in one generation such that the population size for the growth term in the second generation more than compensates.

One challenge to quantifying the degree of the change in total population size (hydra effect) is the sensitivity of the system to initial conditions in chaotic regions of parameter space. For example, when $(\mu ,{\tilde{w}})=(0,0)$, we know mathematically that the total population dynamics of the with-disease system should be identical to that of the disease-free system. However, simulations show that individual trajectories can diverge within 200 generations (Fig. 5). Therefore, we select a threshold at 5% above the disease-free total population size, such that no hydra effect is reported for $r\in [0,320]$ and $d\in [0,1]$ in the $(\mu ,{\tilde{w}})=(0,0)$ using tractable simulation times (See Appendix C). For clarity, we highlight increases in total population size below this threshold, which could be numerical error, by depicting them in gray-scale and larger increases in total population size, in which there is true hydra effect, in a color-scale.

While the degree of hydra effect is independent of initial conditions for large portions of $(\mu ,{\tilde{w}})$ parameter space at fixed *r* and *d* values, there are regions where the degree of hydra effect is strongly dependent on initial conditions. Comparison of these regions to corresponding heat maps of long-time cyclic behavior suggest that these switches are the result of transitions between two (or multiple) alternative long-time behaviors. In this case, the initial condition determines into which basin of attraction the simulation falls. This is supported by systematic variation of a parameter with identical initial conditions, which show clear parameter-induced transitions between long-time behavior structures (Fig. 8).

Discrete-time systems are known to produce complex behaviors at low dimensions (May 1974), but the simple expansion through introduction of infection to a two-dimensional SI system broadens the complexity of dynamics, introducing the appearance of multiple stable structures and a range of behaviors that lead to counter-intuitive changes in total population size. Fundamentally, the observation of these phenomena depend on the overcompensatory nature of the growth term, which potentially leads to increases in population fecundity as a result of decreases in population size. Our work extends understanding of these phenomena, by showing the capacity of infection to produce distinct qualitative long-time behaviors, through both classical and infection-induced hydra effect, which may depend on initial conditions. Thus, this insight provides a means of understanding the complicated behaviors of discrete-time models to a deeper degree.

## Figures and Tables

**Fig. 9:**
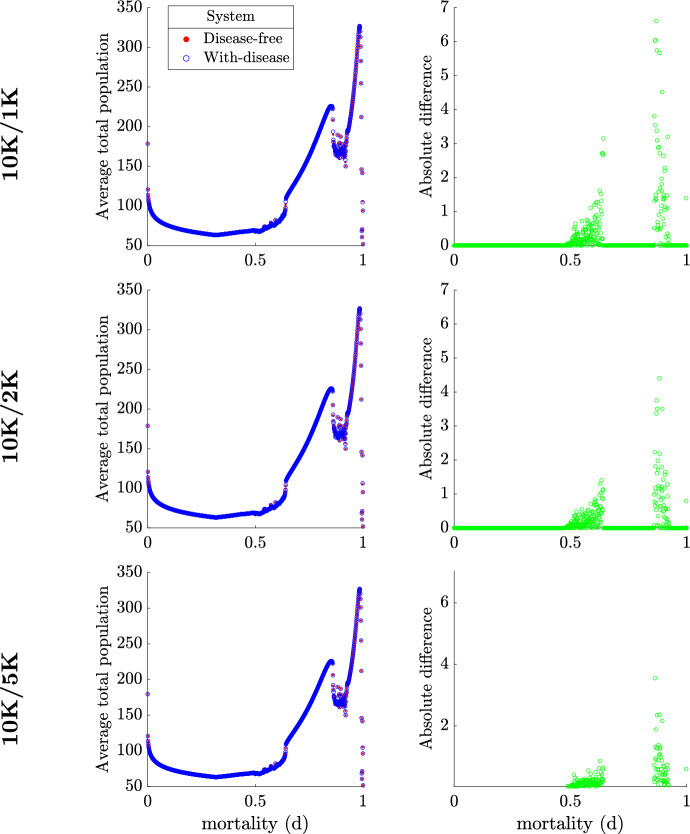
Sensitivity to initial conditions in chaotic regimes creates differences in average population size. *Top row:* 10,000 transients and 1000 generations averaged. *Middle row:* 10,000 transients and 2000 generations averaged. *Bottom row:* 10,000 transients and 5000 generations averaged. *Left column:* Average total population size of the disease-free system (red dots) and the with-disease system (blue circles). *Right column:* Absolute difference between average populations of the disease-free and with-disease systems. Baseline parameters except $r=175$. Standard simulation conditions with $(S_0,I_0)=(150,20)$ (Color figure online)

**Fig. 10:**
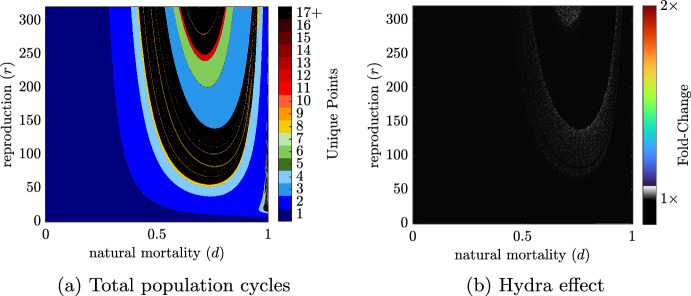
Numerical error induced apparent increase in population size when systems are mathematically equivalent. **a** Length of long-time population cycles of System (1) and **b** population size increase with respect to the parameters *d* and *r* for $(\mu ,{\tilde{w}})=(0,0)$ with all other parameters at baseline values except: $d\in [0:0.001:1]$, $r\in [0:0.5:320]$. For **a**, colorbar: number of unique points in a cycle. For **b**, colorbar: black indicates a reduction in population; gray-scale indicates 0–5% increase in population over the disease-free system; color-scale indicates $>5$% increase in population, represented by fold-change. Standard simulation conditions with $(S_0,I_0)=(150,20)$ (Color figure online)

## Data Availability

The MATLAB codes to generate all necessary simulated data are available on GitHub at https://github.com/laurenchilds/SIdiscrete.

## Appendix A: Detailed simulation methods

Our results are depicted with four distinct figure types. The first three, (1) simulation trajectories (Fig. 5) described in Appendix A.1, (2) long-time behavior (Figs. 3, 8) described in Appendix A.2, and (3) cycle heat maps (Figs. 2, 7) describe in Appendix A.3, are analogous to the simulations, one-, and two-parameter bifurcation diagrams of continuous systems, with one important caveat: the continuation methods used for continuous bifurcation diagrams trace out the behavior of a system across all possible initial conditions. In contrast, our figures are constructed via simulation. Thus they depict the long-time behavior that results from a single set of initial conditions. The fourth type of figure, described in Appendix A.4, considers the change in total population size between the disease-free and with-disease systems (Figs. 4, 6).

### A.1 Simulation trajectories

Long-time simulations were conducted using a specified set of model parameters and initial conditions by updating System (2) for 10,048 generations. The first 10,000 generations were discarded as transients and the final 48 generations were assumed to be representative of the long-time behavior of the system. See Appendix A.2 for an explanation of the choice of 48 generations.

### A.2 Summary of long-time behavior

Scatter plots depict the long-time behavior of a discrete-time system with respect to a chosen parameter as it varies (Fig. 8). Data for the diagrams are collected via a series of simulations in which the value of one parameter is varied across the series while all others, including initial conditions, are held constant. The system was simulated for 10,048 generations and the first 10,000 generations were discarded as transient. The observed population counts during the remaining 48 generations form the data for the figure. Forty-eight generations was chosen to allow for three realizations of each value in a 16-cycle trajectory.

### A.3 Heat maps depicting cyclic behavior

Each cell in the heat maps of cyclic behavior depicts the quantity of distinct points in the long-time behavior of a simulation trajectory for a fixed set of model parameters and initial conditions (Figs. 2, 7). The data is obtained from a series of long-time simulations using a technique identical to that Appendix A.2. After each simulation, the quantity of unique points in the long-time behavior of the system is determined using the unique function in MATLAB after first rounding the data points to seven decimal places. Rounding to seven decimal places was chosen to numerically match points between the disease-free and with-disease systems when $(\mu ,{\tilde{w}})=(0,0)$ and we know the total populations should be equivalent. Trajectories with more than 16 distinct points in their long-time behavior are binned into a single $17+$ category that contains both higher-order cycles and presumably aperiodic or chaotic behavior.

### A.4 Hydra effect diagrams

To measure the capacity of disease to induce a hydra effect in our system, we first calculated the average population size (as described in Appendix A.5). The system was classified as exhibiting hydra effect if the total population of the with-disease system was greater than the corresponding disease-free system. Due to potential numerical errors, particularly in chaotic regions, we show 0–5% increase in population size in gray-scale and greater than 5% increase in color-scale. The 5% threshold was chosen as it was sufficient to avoid spurious detection of the hydra effect in equivalent with-disease and disease-free systems, i.e. for $(\mu ,{\tilde{w}})=(0,0)$, when using 2000 generations to calculate the population averages. This spurious behavior arises out of the sensitivity to initial conditions in the chaotic regions (see Appendix A.5) and can be reduced by increasing the quantity of time points used in the average curve calculations (Fig. 9). While we did observe a further decrease in the differences between averages when increasing the number of points averaged, there were diminishing returns. Thus, 2,000 generations was chosen as a compromise between a sufficient accuracy and reasonable computational time.

### A.5 Average curves

The red and blue average curves in each long-time behavior scatter plot (Fig. 8) were constructed using a series of long-time simulations similar to that in Appendix A.2, except that the system was simulated for 12,000 generations rather that 10,048. For the average curves, the first 10,000 generations were discarded as transient, and the final 2000 generations retained. The data from the 2000 retained generations corresponding to each parameter value were then averaged.

When $(\mu ,{\tilde{w}})=(0,0)$, the total population of the disease-free and with-disease systems are mathematically identical. However, in the chaotic regions, there is sensitivity to initial conditions such that trajectories of the equivalent with-disease and disease-free systems separate (Fig. 5). Increasing the number of points considered in the average reduces the difference in the average total population size between the disease-free and with-disease systems (Fig. 9). As mentioned above in Appendix A.3, the choice of averaging 2,000 generations was a compromise between accuracy and computation time. Furthermore, we choose to show hydra effect below 5% in gray-scale because the 5% threshold provides complete accuracy of the known lack of hydra effect in the $(\mu ,{\tilde{w}})=(0,0)$ case with our choice of averaging across 2000 generations (Fig. 10).

## Appendix B: Justification of analytical results

Here, we provide support for analytical results presented.

### B.1 Derivation of classical hydra effect from a fixed point

The classical definition of a hydra-effect for a single compartment model is

$$\begin{aligned} \frac{\partial T^* }{\partial \alpha } > 0 \end{aligned}$$

for a parameter $\alpha$ which decreases individual fitness and $T^*$ being a fixed point.

For a two-compartment model with fixed point ($S^*$, $I^*$), consider System (4), where $S^*$ and $I^*$ are implicit functions of a parameter $\alpha$. Taking the derivative of the system with respect to $\alpha$, we find

$$\begin{aligned} \begin{pmatrix} \frac{\partial S^*}{\partial \alpha } \\ \frac{\partial I^*}{\partial \alpha } \end{pmatrix}&= \begin{pmatrix} \frac{\partial f_S}{\partial S} &{} \frac{\partial f_S}{\partial I} \\ \frac{\partial f_I}{\partial S} &{} \frac{\partial f_I}{\partial I} \end{pmatrix} \Bigg |_{(S^*,I^*)} \begin{pmatrix} \frac{\partial S^*}{\partial \alpha } \\ \frac{\partial I^*}{\partial \alpha } \end{pmatrix} + \begin{pmatrix} \frac{\partial f_S}{\partial \alpha } \\ \frac{\partial f_I}{\partial \alpha } \end{pmatrix}\Bigg \vert _{(S^*,I^*)} \\&= {\textbf{J}} \Bigg |_{(S^*,I^*)} \begin{pmatrix} \frac{\partial S^*}{\partial \alpha } \\ \frac{\partial I^*}{\partial \alpha } \end{pmatrix} + \begin{pmatrix} \frac{\partial f_S}{\partial \alpha } \\ \frac{\partial f_I}{\partial \alpha } \end{pmatrix}\Bigg |_{(S^*,I^*)} , \end{aligned}$$

where ${\textbf{J}}$ is the Jacobian. Rearranging and solving for $\frac{\partial S^*}{\partial \alpha }$ and $\frac{\partial I^*}{\partial \alpha }$, we find

$$\begin{aligned} \begin{pmatrix} \frac{\partial S^*}{\partial \alpha } \\ \frac{\partial I^*}{\partial \alpha } \end{pmatrix}&= - ({\textbf{J}}-{\textbf{I}}_2) ^{-1}\Bigg |_{(S^*,I^*)} \begin{pmatrix} \frac{\partial f_S}{\partial \alpha } \\ \frac{\partial f_I}{\partial \alpha } \end{pmatrix}\Bigg |_{(S^*,I^*)}\\&= \frac{-1}{|{\textbf{J}}-\mathbf {I_2}|} \begin{pmatrix} \frac{\partial f_I}{\partial I}-1 &{} -\frac{\partial f_S}{\partial I} \\ -\frac{\partial f_I}{\partial S} &{} \frac{\partial f_S}{\partial S} -1 \end{pmatrix} \Bigg |_{(S^*,I^*)} \begin{pmatrix} \frac{\partial f_S}{\partial \alpha } \\ \frac{\partial f_I}{\partial \alpha } \end{pmatrix}\Bigg |_{(S^*,I^*)}\\&= \frac{-1}{|{\textbf{J}}-\mathbf {I_2}|} \begin{pmatrix} \left( \frac{\partial f_I}{\partial I}-1\right) \frac{\partial f_S}{\partial \alpha } -\frac{\partial f_S}{\partial I}\frac{\partial f_I}{\partial \alpha } \\ -\frac{\partial f_I}{\partial S}\frac{\partial f_S}{\partial \alpha } + \left( \frac{\partial f_S}{\partial S} -1\right) \frac{\partial f_I}{\partial \alpha } \end{pmatrix} \Bigg |_{(S^*,I^*)}. \end{aligned}$$

Thus, System (4) exhibits a classical hydra effect with respect to a parameter $\alpha$ if

$$\begin{aligned} \frac{\partial T^*}{\partial \alpha } = \frac{-\left( \left( \frac{\partial f_I}{\partial I}-1\right) \frac{\partial f_S}{\partial \alpha } -\frac{\partial f_S}{\partial I}\frac{\partial f_I}{\partial \alpha } -\frac{\partial f_I}{\partial S}\frac{\partial f_S}{\partial \alpha } + \left( \frac{\partial f_S}{\partial S} -1\right) \frac{\partial f_I}{\partial \alpha }\right) }{|{\textbf{J}}-\mathbf {I_2}|} \Bigg |_{(S^*,I^*)} >0. \end{aligned}$$

### B.2 Restatement and proof of Theorem 1

#### Theorem

(Restatement of Theorem 1) Given System 2 with fixed point $T^*$, if $\ln \left( \frac{r}{d}\right) <\frac{wbd}{\beta (1-d)}$ and there is a hydra effect when $\mu$ is the hydra effect parameter, i.e.,

$$\begin{aligned} \frac{\partial T^*}{\partial \mu }>0, \end{aligned}$$

then there is also a hydra effect when ${\tilde{w}}$ is the hydra effect parameter, i.e.

$$\begin{aligned} \frac{\partial T^*}{\partial {\tilde{w}}}>0. \end{aligned}$$

#### Proof of Theorem 1

Let $f_S^*=f_S(S^*,I^*)$ and $f_I^*=f_I(S^*,I*)$ represent a fixed point solution of System (4) and let ${\bar{\textbf{J}}}$ represent the modified version of the Jacobian given by

$$\begin{aligned} \bar{{\textbf{J}}} = {\textbf{J}}-{\textbf{I}}_2 = \begin{pmatrix} \frac{\partial f_S^*}{\partial S} - 1 &{}\quad \frac{\partial f_S^*}{\partial I} \\ \frac{\partial f_I^*}{\partial S} &{}\quad \frac{\partial f_I^*}{\partial I} - 1\end{pmatrix} = \begin{pmatrix} J_{11} - 1 &{}\quad J_{12} \\ J_{21} &{}\quad J_{22} - 1, \end{pmatrix} \end{aligned}$$

where

$$\begin{aligned} J_{11}&= \left( r-br\left( S^{*}+wI^{*}\right) \right) e^{-b\left( S^{*} + wI^{*} \right) } + \left( 1-d \right) e^{-\beta I^*} \\ J_{12}&= \left( rw-brw\left( S^{*}+wI^{*}\right) \right) e^{-b \left( S^{*} + wI^{*} \right) } -\beta \left( 1-d \right) S^{*}e^{-\beta I^*} \\ J_{21}&= \left( 1-d \right) \left( 1-e^{-\beta I^*} \right) \\ J_{22}&= (1-d)(1-\mu ) + \beta (1-d)S^{*}e^{-\beta I^*}. \end{aligned}$$

Recall that $$w=1-{\tilde{w}}$$.

*Hydra effect with* $\mu$ From our definition of hydra effect, if $\mu$ is the hydra effect parameter, the hydra effect is present at a fixed point if and only if

$$\begin{aligned} \frac{-1}{\left| \bar{{{\textbf {J}}}} \right| } \left( - \frac{\partial f_S^*}{\partial I} + \left( \frac{\partial f_S^*}{\partial S}-1\right) \right) \frac{\partial f_I^*}{\partial \mu } > 0, \end{aligned}$$

since $\frac{\partial f_S^*}{\partial \mu }=0$, which simplifies to

$$\begin{aligned} \frac{1}{\left| \bar{{{\textbf {J}}}} \right| } \left( - \frac{\partial f_S^*}{\partial I} + \frac{\partial f_S^*}{\partial S}-1\right) > 0, \end{aligned}$$

as $$\frac{\partial f_I^*}{\partial \mu }<0$$.

We first focus on the sign of the non-determinant part of our hydra effect condition with the parameter $\mu$, i.e,

$$\begin{aligned} \frac{\partial f_S^*}{\partial S} -1 - \frac{\partial f_S^*}{\partial I} = J_{11} -1 -J_{12}. \end{aligned}$$

To show that this term is always negative, we show that $J_{11} - 1 < J_{12}$. Combining terms and using Lemma 2 (the proof for Lemma 2 in found in Appendix B.3) as well as our condition in Theorem 1, i.e. $\ln \left( \frac{r}{d}\right) <\frac{wbd}{\beta (1-d)}$, we find

$$\begin{aligned} J_{11} - J_{12} - 1&= {\tilde{w}}\left( r - br(S^* + wI^*)\right) e^{-b(S^* + wI^*)} + {\tilde{w}}(1-d)e^{-\beta I^*} \\&\quad +\, w(1-d)e^{-\beta I^*} + \beta (1-d)S^*e^{-\beta I^*} - 1 \\&< {\tilde{w}}+ w(1-d)+\frac{\beta }{b}(1-d)\ln \left( \frac{r}{d}\right) - 1\\&< {\tilde{w}} + w(1-d)+wd -1 \\&= 0. \end{aligned}$$

As $J_{11} - J_{12} - 1 <0$, the sign of this term in the hydra effect condition is negative, and presence of the hydra effect is determined by the sign of the determinant of $\bar{{\textbf{J}}}$, such that hydra effect is determined by

$$\begin{aligned} \frac{1}{\left| \bar{{{\textbf {J}}}} \right| } < 0. \end{aligned}$$

Thus, follows that there is a hydra effect if and only if $\bar{{\textbf{J}}}$ has negative determinant. Thus, we next focus on the sign of $\bar{{\textbf{J}}}$ and thus the signs of the elements of $\bar{{\textbf{J}}}$.

Note that $J_{21}\ge 0$ as $d\in [0,1]$ and $I^*\ge 0$.

Next, we show that $J_{11}-1 < 0$ by showing $J_{11} < 1$. To this end, we divide both sides of the first equation in System (2) evaluated at the fixed point by $S^*$ to obtain

$$\begin{aligned} 1&= re^{-b(S^* + wI^*)} + rw \frac{I^*}{S^*}e^{-b(S^* + wI^*)} + (1-d)e^{-\beta I^*} \end{aligned}$$

and rearrange to get

$$\begin{aligned} 1 - rw \frac{I^*}{S^*}e^{-b(S^* + wI^*)}&= re^{-b(S^* + wI^*)} + (1-d)e^{-\beta I^*}. \end{aligned}$$

The right hand side appears in $J_{11}$, thus

$$\begin{aligned} J_{11}&= re^{-b\left( S^{*} + wI^{*} \right) }-br\left( S^{*}+wI^{*}\right) e^{-b\left( S^{*} + wI^{*} \right) } + \left( 1-d \right) e^{-\beta I^*}, \\&= 1 - rw \frac{I^*}{S^*}e^{-b(S^* + wI^*)}-br\left( S^{*}+wI^{*}\right) e^{-b\left( S^{*} + wI^{*} \right) }, \\&< 1, \end{aligned}$$

because at the endemic equilibrium, $S^*>0$ and $I^*>0$, thus $\frac{I^*}{S^*} > 0$ and $S^*+wI^*>0$, and all parameters are positive. Thus, $J_{11}-1<0$.

Given the condition in Theorem 1, it follows that $\ln \left( \frac{r}{d}\right) <\frac{bd}{\beta (1-d)}$, and using Lemma 2, then

$$\begin{aligned} J_{22}&< 1-d + (1-d)(\beta S^*) e^{-\beta I^*}\\&< 1-d + (1-d)\frac{\beta }{b} \ln \left( \frac{r}{d}\right) , \\&< 1, \end{aligned}$$

meaning that $$J_{22} - 1 < 0$$.

We do not have an explicit bound on $J_{12}$ for guaranteeing $\bar{{\textbf{J}}}$ has negative determinant and thus for the presence of the hydra effect. However, it follows that there is a hydra effect if and only if ${\bar{J}}$ has negative determinant.

*Hydra effect with* ${\tilde{w}}$ From our definition of hydra effect, if ${\tilde{w}}$ is the hydra effect parameter, the hydra effect is present at a fixed point if and only if

$$\begin{aligned} \frac{-1}{\left| {\bar{\textbf{J}}} \right| } \left( \left( \frac{\partial f_I^*}{\partial I}-1\right) - \frac{\partial f_I^*}{\partial S}\right) \frac{\partial f_S^*}{\partial {\tilde{w}}} > 0, \end{aligned}$$

as $\frac{\partial f_I^*}{\partial {\tilde{w}}}=0$. This can be rewritten in terms of the elements of ${\bar{\textbf{J}}}$ as

$$\begin{aligned} \frac{-1}{\left| {\bar{\textbf{J}}} \right| } \left( J_{22}-1 - J_{21}\right) \frac{\partial f_S^*}{\partial {\tilde{w}}} > 0. \end{aligned}$$

As we previously showed $J_{22}-1<0$ and $J_{21}>0$, $J_{22}-1 - J_{21}<0$, and the condition simplifies to

$$\begin{aligned} \frac{1}{\left| {\bar{\textbf{J}}} \right| } \frac{\partial f_S^*}{\partial {\tilde{w}}} > 0. \end{aligned}$$

We consider two cases, depending on the sign of $\frac{\partial f_S^*}{\partial {\tilde{w}}}$.

In the first case, assume that $b(S^* + (1-{\tilde{w}})I^*) < 1$ as from the first equation in System (2), we know that $\frac{\partial {f_S^*}}{\partial {\tilde{w}}} < 0$ if and only if

$$\begin{aligned} b(S^* + (1-{\tilde{w}})I^*) < 1. \end{aligned}$$

Then, if ${\tilde{w}}$ is the hydra effect parameter, the hydra effect is present if and only if

$$\begin{aligned} \frac{1}{\left| {\bar{\textbf{J}}} \right| } < 0. \end{aligned}$$

In this case, we do not have an explicit bound on $J_{12}$ for guaranteeing $\bar{{\textbf{J}}}$ has negative determinant and thus for the presence of the hydra effect. However, it follows that there is a hydra effect if and only if $\bar{{\textbf{J}}}$ has negative determinant. As this is the same condition for the hydra effect with $\mu$ as the parameter, if there is a hydra effect for ${\tilde{w}}$, there is also a hydra effect with $\mu$ under the conditions in Theorem 1 with $\frac{\partial f_S}{\partial {\tilde{w}}} < 0$.

For the second case, we assume $b(S^* + w I^*) > 1$, which implies that $\frac{\partial f_S}{\partial {\tilde{w}}} > 0$. Thus, there is a hydra effect when ${\tilde{w}}$ is the hydra effect parameter if and only if

$$\begin{aligned} \frac{1}{\left| {\bar{\textbf{J}}} \right| } > 0. \end{aligned}$$

We note that $b(S^* + w I^*) > 1$ yields $J_{12}<0$, so $\bar{{{\textbf {J}}}}$ has positive determinant. Therefore, there is a hydra effect when ${\tilde{w}}$ is the hydra effect parameter, but no hydra effect when $\mu$ is the hydra effect parameter, and the statement in Theorem 1 follows. $\square$

Examining the conditions for which $|{\textbf{J}}-{\textbf{I}}_2|< 0$ (where is the Jacobian of System (4) evaluated at the fixed point) we note that given our condition in Theorem 1, a necessary condition for the hydra effect (regardless of whether $\mu$ or ${\tilde{w}}$ are hydra effect parameters) is $J_{12} > 0$. Moreover, a sufficient condition is $J_{22} + J_{21}, J_{11} + J_{21} < 1$.

### B.3 Restatement and proof of Lemma 2

#### Lemma

(Restatement of Lemma 2) Considering System (2), the unique endemic equilibrium $(S^*, I^*)$ satisfies

$$\begin{aligned} S^* + w I^* < {\bar{S}}^*. \end{aligned}$$

#### Proof of Lemma 2

As we are considering Ricker growth, let $f(x) = re^{-bx}$. Since *f* is monotonically decreasing, it is sufficient to show that $d = f({\bar{S}}) < f(S^* + wI^*)$. Recall from system 1 that $d=f({\bar{S}})$ by

$$\begin{aligned} d{\bar{S}}^* = g({\bar{S}}^*) = r{\bar{S}}^* e^{-b{\bar{S}}^*} ={\bar{S}}^* f({\bar{S}}^*). \end{aligned}$$

To this end, we add together the two equations in system (2), which yields that

$$\begin{aligned} T^*&= (S^*+wI^*)f(S^* + wI^*) + (1-d)S^* + (1-d)(1-\mu )I^*,\\&= (S^*+wI^*)f(S^* + wI^*) + (1-d)T^* - \mu (1-d)I^*, \end{aligned}$$

and

$$\begin{aligned} dT^* = f({\bar{S}})T^*&= (S^*+wI^*)f(S^* + wI^*) - \mu (1-d)I^*,\\&< (S^*+wI^*)f(S^* + wI^*). \end{aligned}$$

Since $T^* \ge S^* + wI^*$, we must have that $f({\bar{S}}) < f(S^* + wI^*)$, and $S^* + w I^* < {\bar{S}}$. $\square$

### B.4 Restatement and proof of Lemma 3

#### Lemma

(Restatement of Lemma 3) A fixed point of the System (2) is equivalent to a fixed point of the equation

B1 $$\begin{aligned} \ln \left( 1-Cz\right) = z\big (\ln \left( A+Bz\right) - \ln (1+Dz)-D\ln (1-Cz)\big ), \end{aligned}$$

where

$$\begin{aligned} \begin{aligned} z&:= \frac{\beta I}{b S}, \quad A:= \frac{d}{r},\qquad B:= \frac{b(1-\nu )}{\beta r},\quad C:= \frac{b(1-\nu )}{\beta (1-d)}, \quad D:=\frac{bw}{\beta },\\&\text {and }\quad \nu :=(1-d)(1-\mu ). \end{aligned} \end{aligned}$$

#### Proof of Lemma 3

From our original System (2) consider the simplification where

$$\begin{aligned} x&:=bS,\\ y&:=\beta I, \end{aligned}$$

which gives

B2 $$\begin{aligned} x_{t+1}= & {} r\left( x_t+\frac{bw}{\beta }y_t\right) \textrm{e}^{-\left( x_t+\frac{bw}{\beta }y_t\right) }+(1-d)x_t \textrm{e}^{-y_t},\nonumber \\ y_{t+1}= & {} \frac{\beta (1-d)}{b}x_t\left( 1-\textrm{e}^{-y_t}\right) +(1-d)(1-\mu ) y_t, \end{aligned}$$

Fixed points of System (2), which are equivalent to fixed points of System (B2), must satisfy

B3 $$\begin{aligned} x^*= & {} r\left( x^*+\frac{bw}{\beta }y^*\right) \textrm{e}^{-\left( x^*+\frac{bw}{\beta }y^*\right) }+(1-d)x^* \textrm{e}^{-y^*},\nonumber \\ y^*= & {} \frac{\beta (1-d)}{b}x^*\left( 1-\textrm{e}^{-y^*}\right) +(1-d)(1-\mu ) y^*, \end{aligned}$$

which, by dividing both equations by $x^*$ and setting $z:=\frac{y^*}{x^*}$ simplifies to

B4 $$\begin{aligned}{} & {} e^{-x^*-Dy^*}= \frac{A+Bz}{1+Dz},\nonumber \\{} & {} e^{-y^*} =1-Cz, \end{aligned}$$

where *A*, *B*, *C*, *D*, and $\nu$ are defined in the statement of Lemma 3.

Taking the logarithm of both sides and dividing the first equation by the second gives

$$\begin{aligned} \frac{1+Dz}{z} = \ln \left( \frac{A+Bz}{1+Dz} \right) \ln ^{-1}(1-Cz), \end{aligned}$$

which can be rewritten as

$$\begin{aligned} \ln \left( 1-Cz\right) = z\big (\ln \left( A+Bz\right) - \ln (1+Dz)-D\ln (1-Cz)\big ). \end{aligned}$$

$$\square $$

### B.5 Restatement and proof of Lemma 4

#### Lemma

(Restatement of Lemma 4) The endemic equilibrium (satisfying $I^* > 0$) exists and is unique.

#### Proof of Lemma 4

We first note that (0, 0) is always a solution for (B2). Another disease-free equilibrium is given by $(0,-\ln \left( d/r\right) )$. We now analyze the solutions of the system (B4) for endemic equilibria, noting that each solution uniquely determines $x^*,y^*$ in (B2).

We begin with the case ${\tilde{w}} = 0$. Here, we always have a solution $z=0$. We next observe that the function $f(z):=\ln ({1-Cz})$ is strictly concave in (0, 1/*C*) for $C>0$, while the function $g_0(z):=z\ln (A+Bz)$ is strictly convex in $(0,\infty )$ for $A,B>0$. In particular, $f(z)=g_0(z)$ cannot have more than two solutions. We can show that the second (endemic) solution for $f(z)=g_0(z)$ exists if and only if

B5 $$\begin{aligned} A<e^{-C}. \end{aligned}$$

In particular, if $A<e^{-C}$, we have $f'(0)>g_0'(0)$, and since $f(1/C)=-\infty$ and $g_0(1/C)>-\infty$, the two graphs have to intersect for some $z\in (0,1/C)$. On the other hand, if $A\ge e^{-C}$, then it follows from $f''<0$, $g_0''>0$ on [0, 1/*C*) that $f'(z)<g_0'(z)$ for all $z\in (0,1/C)$, and so $f(z)<g_0(z)$ as well. Thus the two graphs intersect only at 0.

The same argument shows that the condition (B5) persists for ${{\tilde{w}}}>0$ as well, provided the function

$$\begin{aligned} g_{{\tilde{w}}}(z):=z\left( \ln \left( A+Bz\right) -\ln \left( 1+{\tilde{w}}z\right) -{\tilde{w}}\ln (1-Cz)\right) \end{aligned}$$

is strictly convex in (0, 1/*C*). This is true for all $C\ge 3$, for example, which is sufficient for our purposes. $\square$

### B.6 Restatement and proof of Theorem 5

#### Theorem

(Restatement of Theorem 5) Given a fixed point $(S^*,I^*)$ for System (2), which is equivalent to a fixed point $z^*$ for the Eq. (B1), sufficient conditions to guarantee an increase in population size when infection is introduced (infection-induced hydra effect) are

$$\begin{aligned} w < 1 - \frac{\beta }{b} \quad \textrm{and} \quad d\ge \frac{1}{2}. \end{aligned}$$

We note that the existence of the hydra effect is independent of $\mu$.

#### Proof of Theorem 5

To begin, consider the simplified System (B4), which assumes the existence of an endemic equilibrium. Raise the second equation to power $\frac{b}{\beta }(1-w)$ and then multiply together the left hand sides and right hand sides of these equations to obtain

$$\begin{aligned} e^{-x^*-Dy^*}\cdot e^{-\frac{b}{\beta }(1-w)y^*}= \frac{A+Bz}{1+Dz}(1-Cz)^{\frac{b}{\beta }(1-w)}, \end{aligned}$$

which simplifies to

B6 $$\begin{aligned} e^{-x^*-\frac{b}{\beta }y^*}= \frac{A+Bz}{1+Dz}(1-Cz)^{\frac{b}{\beta }(1-w)}. \end{aligned}$$

Notice that the left hand side is equivalent to $e^{-b(S^*+I^*)}$.

Recall, that we want conditions for which the endemic equilibrium is larger than the disease-free equilibrium, which is given by System (B4) when $y^*=\beta I^* = 0$. This reduces to

$$\begin{aligned} e^{-{\bar{x}}^*} = A, \end{aligned}$$

where ${\bar{x}}=b{\bar{S}}$, refers to the solution to the disease-free system.

Thus, we want the right hand side of Eq. (B6) to be smaller than $e^{-{\bar{x}}^*}=A$, which would imply $-bS^*-bI^* < -b {\bar{S}}^*$ and, thus ${\bar{S}}^* < S^*+I^*$. This represents an increase in total population size with infection, or hydra effect. Thus, we want

$$\begin{aligned} e^{-b(S^*+I^*)}= e^{-x^*-\frac{b}{\beta }y^*}= \frac{A+Bz}{1+Dz}(1-Cz)^{\frac{b}{\beta }(1-w)}< A. \end{aligned}$$

Focusing on the right most inequality, this is equivalent to

$$\begin{aligned} \left( 1+ \frac{B}{A}z\right) (1-Cz)^{\frac{b}{\beta }(1-w)}<1+Dz. \end{aligned}$$

For this inequality to hold, it is sufficient to have $\frac{b}{\beta }(1-w)>1$ and $\frac{B}{A}\le C$, because

$$\begin{aligned} \left( 1+ \frac{B}{A}z\right) (1-Cz)^{\frac{b}{\beta }(1-w)}&< \left( 1+ Cz\right) (1-Cz)^{\frac{b}{\beta }(1-w)},\\&< \left( 1+ Cz\right) \frac{(1-Cz)}{(1-Cz)}(1-Cz)^{\frac{b}{\beta }(1-w)},\\&< \left( 1 - C^2z^2\right) (1-Cz)^{\frac{b}{\beta }(1-w)-1},\\&<1, <1+Dz.\\ \end{aligned}$$

The first condition, $\frac{b}{\beta }(1-w)>1$, simplifies to $w < 1- \frac{\beta }{b}$. The second condition, $\frac{B}{A}\le C$, simplifies to $d\ge 1-d$. As $d\in [0,1]$, this implies $d\ge \frac{1}{2}$. $\square$

## Appendix C: Supplemental results

See Appendix Figs. 9 and 10.

## Declarations

### Code availability

The MATLAB codes are available on GitHub at https://github.com/laurenchilds/SIdiscrete.
